# Reciprocal semantic predictions drive categorization of scene contexts and objects even when they are separate

**DOI:** 10.1038/s41598-020-65158-y

**Published:** 2020-05-21

**Authors:** Anaïs Leroy, Sylvane Faure, Sara Spotorno

**Affiliations:** 1 Laboratoire d’Anthropologie et de Psychologie Cliniques, Cognitives et Sociales (LAPCOS), Université Cote d’Azur, Nice, France; 2 School of Psychology, University of Keele, Keele, United Kingdom

**Keywords:** Visual system, Human behaviour

## Abstract

Visual categorization improves when object-context associations in scenes are semantically consistent, thus predictable from schemas stored in long-term memory. However, it is unclear whether this is due to differences in early perceptual processing, in matching of memory representations or in later stages of response selection. We tested these three concurrent explanations across five experiments. At each trial, participants had to categorize a scene context and an object briefly presented within the same image (Experiment 1), or separately in simultaneous images (Experiments 2–5). We analyzed unilateral (Experiments 1, 3) and bilateral presentations (Experiments 2, 4, 5), and presentations on the screen’s horizontal midline (Experiments 1–2) and in the upper and lower visual fields (Experiments 3, 4). In all the experiments, we found a semantic consistency advantage for both context categorization and object categorization. This shows that the memory for object-context semantic associations is activated regardless of whether these two scene components are integrated in the same percept. Our study suggests that the facilitation effect of semantic consistency on categorization occurs at the stage of matching the percept with previous knowledge, supporting the object selection account and extending this framework to an object-context reciprocal influence on matching processes (object-context selection account).

## Introduction

Viewers are able to categorize visual images from a brief glance, in a few dozens of milliseconds^1^. This ability is a central dimension of cognition^2^: We make sense of the world fundamentally by relating and differentiating its components in terms of known groups, or categories^3,4^. This allows us to process large amounts of information, while minimizing cognitive costs, and perceive common structures of elements^4^, even within the complex scenes that characterize everyday visual world. These scenes are composed of multiple objects, organized within a background (the scene’s context) following semantic and spatial rules, which define the plausibility of object occurrence in the scene and of the scene’s layout, respectively^5^. Object-context associations are at the core of the concept of “scene” and a key, omnipresent aspect in our visual experience. However, it is still unclear how they influence visual information processing, namely in which conditions and at what stage they operate.

Many studies have focused on the plausibility of object occurrence and have shown better categorization performance in the case of consistent, expected associations than inconsistent, unexpected associations. This semantic consistency advantage has been mainly found for categorization of the objects (e.g., “parasol”, “notebook”) included in the scene^6–10^ but also for the categorization of the scene’s context^7,11,12^ in terms of its gist^13^ (e.g., “beach”, “office”).

Several studies using even-related potentials (ERPs)^14–18^ have reliably reported a greater N400 evoked by inconsistent objects, and interpreted it as reflecting difficulties in semantic object-context integration, similarly to what happens with semantic violations during language processing. They also found, albeit not always^14^, that object-context inconsistency produces earlier ERP modulations (e.g., N300), even though it is still not clear whether they are truly distinct from the later modulations^19^ and, if this is the case, whether they are pre-semantic or indicate ongoing semantic processing leading to progressively refine object recognition^15–17^. Proposed explanations of the consistency effect are, thus, contradictory, and situate it on a different time scale. Moreover, they focus on how the scene’s context influences categorization of the embedded objects, without considering any impact of the objects on context categorization.

Two accounts posit that we use predictions of expected (consistent) semantic associations to guide visual information gathering and that, therefore, object-context associations influence directly the categorization process.

The perceptual schema model (or description enhancement model)^5,20,21^ argues that semantic predictions impact the initial stages of perceptual analysis, before any recognition of the object has begun. These predictions would facilitate, when an object is expected in a given scene’s context, the extraction of the object’s visual features and the construction of a structured visual description of the object (acting, therefore, also on figure/ground segregation processes). This would enhance categorization of consistent objects, while perhaps impairing categorization of inconsistent objects. These mechanisms are thought to be based on early inferences of the scene’s gist based on coarse, low spatial frequency (LSF) information^6^, which would also enable activation of long-term knowledge related to the scene (schema), containing indications about what objects to expect.

The object selection model (or priming model^6,8,16,17,22,23^) also proposes that the categorization of the scene’s context influences that of the embedded objects, but at a later stage, after the construction of an object’s visual description. Instead of affecting perceptual analysis, it would affect the viewer’s criterion, i.e., how much perceptual evidence is needed to access the object’s category by matching the perceived object with the object representation(s) selected in long-term memory. The matching process would start from a partial recognition of the object from coarse information (of a structured percept, segregated with respect to the background, although lacking detail), which would activate a set of plausible representations. Semantically consistent object-context associations would facilitate object categorization by lowering the quantity of evidence required for a satisfactory matching.

A third account, the functional isolation model^24,25^, claims that the scene’s context has no effect on object categorization *per se*. Rather, contexts and objects are processed independently and integration of information takes place at a post-perceptual, decision-making stage of response selection. Any (apparent) facilitation of semantic consistency on categorization would come from an experimental bias promoting guessing responses. For instance, Biederman and colleagues^5^ presented the name of an object followed by a scene and asked participants to judge whether the object occurred in the scene. Hollingworth and Henderson^24^ argued that cueing the target object’s identity before the scene preactivates expectations and leads viewers to be more likely to respond positively about object occurrence within a consistent than an inconsistent context, and this even when the object is absent. More false alarms were indeed found in the consistent condition compared to the inconsistent condition in Biederman *et al*.’s. However, the two conditions when computing the sensitivity score (*d’*) were pooled together, consequently overestimating the sensitivity rate in the case of consistency and underestimating it in the case of inconsistency. When Hollingworth and Henderson reran the study computing *d’* after separating false alarms for the consistent and inconsistent trials, or presenting first the scene and then two objects between which to choose the one included in the scene, no semantic consistency advantage was reported.

**Figure 1:**
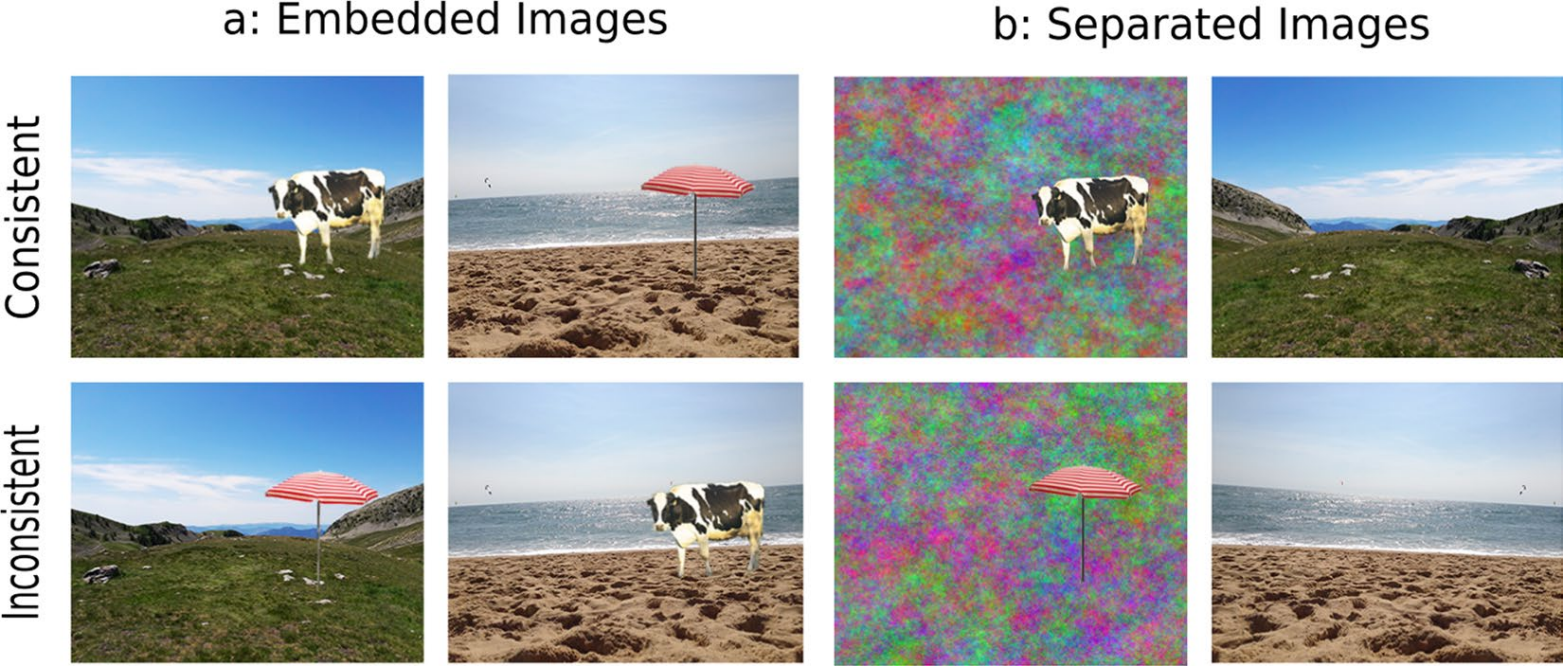
Examples of semantically consistent (top row) and inconsistent (bottom row) presentations, when either the object was included into the scene’s context (**a**: Experiment 1) or the object and the context were shown simultaneously but separately, with the object pasted onto a 1/*f* colored noise background (**b**: Experiments 2–5). The images of the mountain and of the parasol were adapted from photos taken by the first author, while the images of the beach and of the cow were adapted from photos shared in Creative Commons, under a CC BY-SA 2.0 license (https://creativecommons.org/licenses/by-sa/2.0/?ref=ccsearch&atype=rich) for the beach and a CC BY 2.0 license (https://creativecommons.org/licenses/by/2.0/?ref=ccsearch&atype=rich) for the cow. The image of the beach can be found at this link: https://search.creativecommons.org/photos/8668bad5-87ab-471b-a392-888595f28e96. The only change to this image was a mirror reversal to illustrate the visual presentation in each hemifield (see General Method for more details). The image of the cow have been cropped, and mirror reversed, from a larger picture which can be found at this link: https://search.creativecommons.org/photos/864c293f-7e2f-4ff8-b4be-9a76b261fc61. These images were not used as experimental materials and are presented here only for illustrative purposes.

The general aim of the present study was to improve understanding of the object-context interplay in visual scene processing. For this purpose, we manipulated the semantic association (consistent versus inconsistent) between the scene’s contexts and the objects (Fig. 1) in five experiments using fast presentations and asking participants to categorize both the context and the object.

Our main objective was to test predictions from the three theories described above, and to do so we introduced three fundamental aspects in our procedure (see General Method for more details).

**Figure 2:**
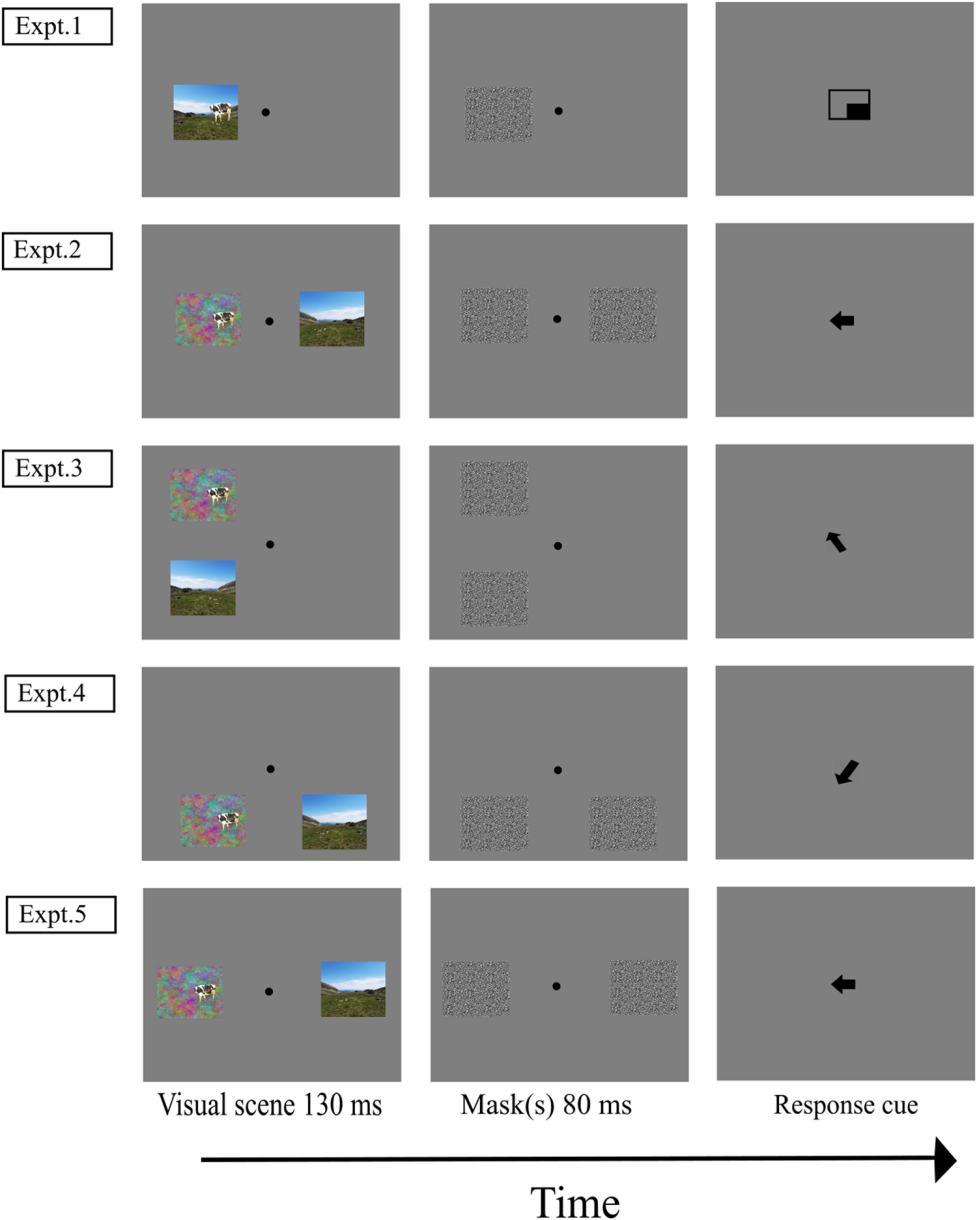
Time course of a trial in each experiment. The trial started with a single-point calibration check and a 90-ms black fixation cross (both in the center of the screen and here not depicted). Then, one image depicting an object included into a scene’s context (Experiment1) or one object image and one context image (Experiments 2–5) were briefly presented and then masked. After this, a 150-ms medium gray blank screen was presented (here not depicted) and then a response cue (until response) indicated whether the participant had to categorize the object or the context first. Please note that the depictions here do not respect the original size proportions of stimuli presentation and that these images were not used as experimental materials and are presented here only for illustrative purposes. The image of mountain was adapted from photo taken by the first author. The image of the cow have been cropped from a larger picture shared under a CC BY 2.0 license (https://creativecommons.org/licenses/by/2.0/?ref=ccsearch&atype=rich) and can be found at this link: https://search.creativecommons.org/photos/864c293f-7e2f-4ff8-b4be-9a76b261fc61.

First, we varied how object and context images were presented (Fig. 2). In Experiment 1, the objects were embedded into the scene’s contexts, to set a baseline for the consistency effect in our study. In all the other experiments, one object image and one context image were shown simultaneously but separately in different locations in the visual field, with the object embedded into a 1/*f* colored noise background (Fig. 1, third column) reproducing the spatial frequency distribution of a natural scene. We used these separate and simultaneous presentations as a key manipulation to examine in what measure any semantic consistency effect requires low-level, perceptual relationships between the context and the object within the same image, or may arise exclusively from high-level guidance, independently from the actual and reciprocal organization of the context and the object in a unique percept. Moreover, and importantly, the simultaneous image presentation avoided any possible bias related to a priming effect (semantic preactivation) which may occur with sequential presentations (see also e.g.,^7,15,16^). Previous research has reported a semantic consistency categorization advantage for separate and simultaneous object images^26,27^, even after correcting for response bias, which appeared to explain only part of the effect^28^. To our knowledge, no study has analyzed the semantic consistency effect between an object and a scene’s context (background) with separate and simultaneous presentations.

Second, we did not constrain response within a preselected range of options. Consequently, the probability to guess correctly was arguably weak, as participants could choose from virtually unlimited labels at the entry-level category^7,10^. Thus, even when they correctly recognized one of the images (e.g., a farm) and gave then a response for the other image guessing on the basis of a semantic association, they were quite unlikely to give the correct answer (e.g., they could respond “pig”, “goose”, etc., instead of the correct label “cow”).

Third, and again to minimize any impact of a guessing strategy, we removed from our analyses any trial with an intrusion response, which is an incorrect response given for one image and semantically coherent with the co-occurring image, for which the response has been correct^7^. For instance, when presented with the images of Fig. 1, participants might correctly recognize the mountain (context) and then guess the other image might be a goat, while it is a cow or a parasol, or they might correctly recognize the cow and then guess that the context is a farm, while it is a mountain or a beach. In addition, semantically associated responses might occur even when both the response for the context and that for the object are incorrect. Therefore, we also computed the number of consistent but both incorrect responses.

### Different outcomes would support different theories

According to the perceptual schema model, semantics influence the early stage of object’s perceptual analysis and, thus, the extraction of the object from the scene’s context. Thus, we expected a semantic consistency effect only in Experiment 1, where the object was included into the scene’s context. Moreover, we expected the effect only for object categorization: this model does not easily account for any effect of object processing on context categorization, because the semantic effect would arise from gathering coarse information about the context, which would then affect processing of the local (object) information within the image.

According to the object selection model, the semantic consistency effect should occur in all our experiments and facilitate categorization of both the object and the context. In the classic version of this model, different possible perceptual representations of the same object are first accessed from coarse (contextual) information, and then the progressive accumulation of finer information enables selection of the best matching one^29^. However, we hypothesized that the same account may apply to the influence of objects on context categorization, as object processing may activate knowledge about the scene’s context where the object is likely to appear^6^, lowering the viewer’s criterion in matching the perceived context with a context representation in the case of semantic consistency.

Finally, according to the functional isolation model, we expected no semantic consistency effect because of the precautions (see above) taken in our study to limit the influence of a guessing strategy favoring performance in the consistent condition. Moreover, this model would predict many intrusion responses and many cases with two consistent but incorrect responses for the object-context pairs.

Besides providing a fundamental test for the theories concerning the impact of object-context semantic associations on visual categorization, our study offers other theoretical insights by examining categorization performance for both scenes’ contexts and objects, and presenting the images lateralized in the left and/or right visual field (whereas previous research has used central presentations). In this way, we could analyze whether both context and object image levels can be quickly categorized without the contribution of foveal (central, high acuity^30^) vision, and at different eccentricities (see General Method), therefore with limited involvement of detailed perceptual information (high spatial frequencies, HSF). Moreover, we could examine any impact of such extrafoveal presentations and of varying eccentricity on the semantic consistency effect for both contexts and objects.

## Results

Participants had to name both the context and the object as accurately as possible. They were instructed to be as specific as possible (e.g., “priest” rather than “person”, “lounge” rather than “house”), as in^7^. The categorization order was counterbalanced between participants and reminded them with a cue (see Fig. 2) indicating where the image they had to categorize first appeared. For each experiment, we ran a generalized linear mixed model (GLMM) on accuracy with Semantic Association (Consistent, Inconsistent), Image Type (Context, Object) and their interaction as predictors, and participants and images as random factors. All GLMMs had full random structure. For comparison between the experiments, we used independent-sample *t* tests. Prior to analysis, we removed the trials where participants gave incorrect responses classifiable as intrusions (Table 1). We present the results obtained after these removals, but we found the same patterns of results in each experiment also when including the trials with intrusions (see Supplementary Materials: Table S1 for accuracy on overall trials, with and without intrusions; Table S2 for corrected accuracy, i.e., hit rate minus intrusion rate, considering any intrusion as a false alarm, as in^7^). Importantly, and despite the fact that we asked participants to categorize both the context and object, the amount of intrusions was overall very low (*M* = 2.82%, *SD* = 2.05), suggesting little use of any strategy to guess semantically consistent responses.

**Table 1:** Number and percentage (in brackets) of trials removed prior to analysis, for each experiment.

Number (%) of trials with:	Experiment				
	1	2	3	4	5
- no reliable central fixation at image onset	35 (2.8)	45 (4.3)	24 (1.9)	58 (4.6)	34 (2.0)
- intrusion responses	144 (6.3)	79 (3.4)	18 (0.7)	29 (1.2)	76 (3.0)
- two incorrect but consistent responses	105 (4.1)	46 (1.8)	18 (0.7)	47 (1.8)	60 (2.3)

Top row: trials with no reliable central fixation at experimental image onset (error in the single-point calibration check was >1° or distance of gaze position from the screen’s center was >1°); percentages computed on the overall data. Middle row: trials with intrusion responses (i.e., with one incorrect response that was semantically consistent with the other, correct one); percentages computed on the data after the removals of trials with no central fixation at image onset. Bottom row: trials with two incorrect responses that were semantically consistent with each other; percentages computed on the data after the removals of trials with no central fixation at experimental image onset.

## Experiment 1

We presented the context and the object embedded in the same scene (Fig. 2) to examine whether the semantic consistency advantage found in the literature for both context categorization and object categorization replicated with our set of stimuli and with lateralized presentations, and to establish a baseline for the consistency effect in our study.

**Figure 3:**
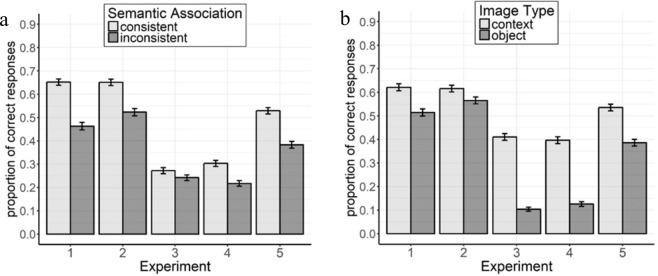
Categorization accuracy in Experiments 1–5 as a function of Semantic Association (Consistent vs. Inconsistent; **a**) and of Image Type (Context vs. Object; **b**). Error bars indicate one standard error around the mean.

We found a main effect of Semantic Association (Fig. 3a), *β* = −1.289, *SE* = 0.227, *z* = 5.69, *p* < 0.001, as images semantically consistent were categorized more accurately (*M* = 0.65) than inconsistent ones (*M* = 0.46), and of Image Type (Fig. 3b), *β* = −0.736, *SE* = 0.258, *z* = −2.85*, p* = 0.004, as the context was categorized better (*M* = 0.62) than the object (*M* = 0.51). The interaction was not significant, *β* = −0.015, *SE* = 0.340, *z* < 1, *p* = 0.966.

The advantage of semantic consistency for the categorization of both the context and the object (comparable for the two image types) is in agreement with several studies using central image presentation^7,9–11^. We can, thus, conclude that our first experiment replicated previous findings and extended them to lateralized images perceived extrafoveally.

An important limitation of this approach is that having one level (the object) nested into the other (the context) does not allow us to disentangle, within the semantic consistency effect, the impact of low-level, perceptual relationships between the context and the object from that of high-level, cognitive guidance and representation. To examine this issue, Experiment 2 presented the context and the object separately, each in a different visual field with respect to the vertical midline (Fig. 2).

## Experiment 2

We found a main effect of Semantic Association, *β* = −0.875, *SE* = 0.228, *z* = −3.83*, p* < 0.001, (Fig. 3a) as semantically consistent images were better categorized (*M* = 0.65) than inconsistent ones (*M* = 0.52). The main effect of Image Type, *β* = −0.375, *SE* = 0.271, *z* = −1.38, *p* = 0.166, (Fig. 3b) and the interaction, *β* = −0.200, *SE* = 0.431, *z* < 1, *p* = 0.642, were not significant.

The consistency advantage clearly shows that viewers used semantic predictions about the likely semantic associations within scenes to guide categorization of both contexts and objects in a similar way, even when these two scene components do not perceptually belong to the same image. This facilitation, moreover, was comparable to when the context and the object belonged to the same image: the mean accuracy difference between semantically consistent and inconsistent trials did not significantly differ in Experiment 1 (0.19) vs. Experiment 2 (0.13), *t*(30)=1.85, *p* = 0.076. These findings suggest that the influence of semantic consistency acts at a representational level, rather than at an early perceptual level concerning the extraction of the visual features of the stimulus (namely, of the local object from the global scene’s context)^5,20,21^. As this aspect is crucial for our study, we will address it in detail in the General Discussion.

The absence of differences in categorization accuracy between context and object images seemed to arise from a slightly better performance for the objects in Experiment 2 (*M* = 0.57) than in Experiment 1 (*M* = 0.51), when they were included in the scene’s context (although this difference was not significant, *t*(30)=1.09, *p* = 0.285; Cohen’s *d* = 0.039), while accuracy for context categorization was virtually the same between these two experiments (both *Ms* = 0.62, *t*(30)<1, *p* = 0.891). Previous studies^7,31^ have reported an advantage for isolated objects compared to objects included within scene’s contexts. However, in previous work the advantage might have arisen from easier figure/ground segregation, as the isolated objects were pasted into a blank background. In our experiment the objects were embedded into 1/f noise backgrounds, which reduced, even though maybe not completely removed, this problem. We speculate, therefore, that the relative facilitation of object categorization in Experiment 2 may rather mainly stem from how we presented the simultaneous images. The short and separate presentation of the object and the context each in a different hemifield directed each image exclusively to the contralateral cerebral hemisphere^32^. Therefore, initial information processing was divided between the two hemispheres, instead of taxing the same hemisphere as in Experiment 1. Moreover, the advantage of separate, bilateral presentations is known to emerge mainly for the most difficult task condition^33,34^, here object categorization.

To (a) generalize the semantic consistency effect between separate images to different views in the visual field and (b) to differentiate the impact of a separate presentation of the object and the context from the impact of a unilateral vs. bilateral presentation on object categorization, Experiment 3 presented context and object images separately, as Experiment 2, but within the same hemifield (left or right), thus both initially directed to the same hemisphere.

## Experiment 3

We found a main effect of Semantic Association, *β* = −0.379, *SE* = 0.176, *z* = −2.15, *p* = 0.031, (Fig. 3a) with better accuracy for consistent (*M* = 0.27) than inconsistent (*M* = 0.24) images, and of Image Type, *β* = −2.599, *SE* = 0.355, *z* = −7.31, *p* < 0.001, (Fig. 3b) as contexts were better categorized (*M* = 0.41) than objects (*M* = 0.10). The interaction was not significant, *β* = −0.506, *SE* = 0.348, *z* = −1.46, *p* = 0.145.

The essential finding is that the advantage for semantically consistent object-context pairings that do not belong to the same perceptual configuration was replicated. Moreover, this effect was comparable for context and object images, as in Experiments 1 and 2, showing that it acts similarly when categorizing either global or more local image regions. Thus, we demonstrated that the consistency effect is not parochial to a specific image location, even though its amount may be modulated by the image arrangement in the visual field. We argue that the smaller difference between consistent and inconsistent trials compared to Experiment 2, *t*(30) = 4.72, *p* < 0.001 (and, by extension, Experiment 1) is likely to be due to a performance drop. Indeed, accuracy was much lower in Experiment 3 than 2 (and 1), and for both the context and (strikingly so) the object images (Fig. 3b), both *ts*(30) ≤ 5.19, *p* < 0.001. There are two plausible explanations for this.

The first possibility arises from the unilateral presentation of two images that may cause an overload of the contralateral hemisphere, whereas in Experiment 1 only one image at the time was presented, and in Experiment 2 the two images were presented in an inter-hemifield condition, sharing the (initial) processing costs between the hemispheres. Previous research has suggested that independent, parallel processing of information in inter-hemifield presentations doubles visual short-term memory capacity compared to intra-hemifield presentations^35^.

The second possibility stems from lower spatial resolution and contrast sensitivity, which may be due to the greater overall image eccentricity in Experiment 3 than in Experiments 1–2 (see Procedure): While this was required to ensure lateralized processing, visual acuity decreases the further away from central vision^30^. Moreover, the horizontal–vertical anisotropy^36,37^ must be considered: Vertical eccentricities (greater in Experiment 3 than Experiments 1–2) are more disruptive for spatial resolution, contrast sensitivity^38^, and information accumulation over time^39^ than horizontal eccentricities.

We then examined whether and how the semantic consistency effect between two separate images further generalizes to various arrangements in the visual field, while tackling in a systematic way the two possible explanations of intra- vs. inter-hemispheric processing and of decreased spatial resolution and contrast sensitivity. In Experiment 4, the two images were presented lateralized (as in Experiment 2) but both in the upper or in the lower visual field (as in Experiment 3). Comparing Experiments 3 and 4 allowed us to isolate the effect of intra- vs. inter-hemifield presentation, while keeping constant any other influence in our consistency vs. inconsistency categorization results.

## Experiment 4

We found a main effect of Semantic Association, *β* = −0.736, *SE* = 0.164, *z* = −4.50, *p* < 0.001, (Fig. 3a) with better accuracy for consistent (*M* = 0.30) than inconsistent (*M* = 0.21) images, and of Image Type, *β* = −2.248, *SE* = 0.316, *z* = −7.12, *p* < 0.001, (Fig. 3b) as contexts were better categorized (*M* = 0.40) than objects (*M* = 0.13). The interaction was not significant, *β* = −0.235, *SE* = 0.346, *z* < 1, *p* = 0.450.

Apart from (crucially) finding again the advantage for consistent over inconsistent trials for both objects and contexts, as in the Experiments 1–3, the results are similar to those of Experiment 3 when considering accuracy for context images, *t*(30) < 1, *p* = 0.687, and object images, *t*(30) < 1, *p* = 0.482. This may suggest that the performance drop in Experiment 3 compared to Experiment 2 was mainly due to decreased spatial resolution and contrast sensitivity, rather than to the overload of a single hemisphere from the processing of two simultaneous images. Moreover, a categorization impairment due to decreased spatial resolution and contrast sensitivity would lead to a bigger impact on object categorization (involving more local, detailed information) than on context categorization (based mainly on global, coarse properties), and this is exactly what we reported in Experiments 3–4.

The next and final experiment aimed to tease apart the role of overall image eccentricity from the screen’s center and of horizontal-vertical anisotropy in the context vs. object effect and in any modulation of the influence of semantic consistency on categorization. To this purpose, we presented the two images centered on the horizontal midline, one to the left and the other to the right as in Experiment 2 (reducing any impact of vertical eccentricity), but with an overall image eccentricity that matched that in Experiments 3–4.

## Experiment 5

We found a main effect of Semantic Association, *β* = −0.944, *SE* = 0.183, *z* = −5.14, *p* < 0.001 (Fig. 3a), with an advantage for consistent (*M* = 0.53) compared to inconsistent (*M* = 0.38) images that was similar to the one in Experiment 2, *t*(30)<1, *p* = 0.594. We found a main effect of Image Type, *β* = −0.993, *SE* = 0.259, *z* = −3.84, *p* < 0.001, with better categorization for contexts (*M* = 0.53) than objects (*M* = 0.39) (Fig. 3b). The interaction was not significant, *β* = −0.307, *SE* = 0.333, *z* < 1, *p* = 0.360.

The replication of the semantic consistency advantage for both context and object images also in this experiment shows the robustness of our principal finding.

Experiment 5 differed from Experiment 2 only in the greater overall image eccentricity. Greater eccentricity should logically result in a steeper decrease in categorization accuracy for objects than contexts (finer vs. coarse processing, respectively), and we did show such a differential impact: Accuracy was lower in Experiment 5 than in Experiment 2 for objects, *t*(30)=−3.62, *p* = 0.001, and only tended to be lower for contexts, *t*(30) = −2.02, *p* = 0.053.

However, accuracy in Experiment 5 was higher than in Experiment 3 (+0.28 for objects, +0.12 for contexts; both *ts*(30) ≥ 3.65, *p* < 0.001) and in Experiment 4 (+0.26 for objects, +0.14 for contexts; both *ts*(30) ≥ 3.98, *p* < 0.001), which presented the images at the same overall eccentricity, but in the upper and lower quadrants. These improvements show the importance of differential modulation of categorization due to eccentricity in the vertical compared to the horizontal axis, especially for object categorization, for which, thus, the reduction in spatial resolution and contrast sensitivity associated with greater vertical eccentricity^30^ seems to have a bigger detrimental impact.

## General Discussion

We studied rapid visual categorization comparing systematically performance for objects and scene’s contexts. We focused on the role of object and context associations in scene processing, in terms of semantic expectations arising from previous experience regarding their co-occurrence. We tested predictions from the three principal theories (perceptual schema model, object selection model, functional isolation model) that have been used to account for the well-known, but still highly debated, finding of better categorization with semantically consistent (i.e., plausible) than inconsistent associations^5,7–11^. To do so, in five experiments, we examined whether semantic consistency impacts categorization only when the context and object are integrated in a unique image, as it has exclusively been studied, or even when the perceptual scene configuration is not present in the visual field but only mentally constructed by the viewer.

Overall, showing a consistency advantage when an object image and a context image were presented simultaneously but separately, our study strongly suggests that a scene mental representation does not require a unified scene percept to influence categorization. We reported better categorization in the case of semantically consistent image pairs regardless of the specific image spatial arrangement in the visual field: When they were presented one in the left and one in the right hemifield, along the screen horizontal midline (at smaller, Experiment 2, or greater eccentricity, Experiment 5) or both in the upper or lower quadrants (Experiment 4), and when they were presented one in the upper and one in the lower quadrant in the same hemifield (left or right, Experiment 3). The replication of the consistency advantage, albeit reduced, even in Experiments 3 and 4, where performance was overall decreased with very low accuracy for object images, shows the robustness of our results.

Our study also shows that the object (local) level and the context (global) level influence each other^7,11^, and that the learnt semantic associations between them impact their processing in a similar way. Indeed, we never found an interaction between the semantic consistency and type of image, object or context, categorized.

Both the influence of object-context semantic associations when the object is not embedded in the scene’s context and the mutual interplay between the object and the context argue against the key assumption of the perceptual schema model^5,20,21^ that semantic expectations act directly on the early perceptual processing of the object’s features and on the construction of the object’s structured visual description, allowing the differentiation of the object from its surroundings (the scene’s context).

Our results are also in disagreement with the functional isolation model^24,25^, which proposes that any inconsistency effect is an epiphenomenon arising only during response selection, once all the stages of image categorization (perceptual processing and matching with a relevant long-term-memory representation) have been completed for the object and the context independently. Moreover, according to this model, the consistency advantage in response selection would be due to a guessing strategy promoted by an experimental bias^24^. In our study, as responses were not restricted to a limited, preselected range of options, participants were unlikely to guess the correct answer. We also controlled for any guessing strategy by removing from analysis trials with intrusion (one response correct and the other incorrect but consistent). Few intrusions were made overall, and incorrect but semantically related responses for both the context and object images were rarely given (see Table 1): This suggests that our participants did not tend to guess.

The semantic consistency advantage we showed supports the object selection model^6,8,16,17,22,23^, as it is compatible with the idea that object-context semantic associations influence the stage of matching the percept with previous knowledge and, therefore, with the representation of its category. Moreover, this model does not only account for the consistency effect on object categorization, but it may be extended to explain the reciprocal influence between objects and contexts we found, as the impact of semantic associations on the percept-to-representation matching mechanisms may apply to both the object and context levels. Inspired by the work of Bar and colleagues on object recognition within scene’s contexts^6,29,40^, we suggest that a first, coarse (LSF) percept is processed to a certain extent to allow partial recognition and used to generate a set of predictions, possibly with parallel processing, about the likely representations of the stimuli based on their general structure (object’s silhouette, context’s spatial statistics)^41^. Then, viewers use knowledge of object-context co-occurrence within a type of scene to validate and refine interactively these predictions and, finally, identify the stimuli with the gradual integration of perceptual details (HSF)^42^.

The overall superiority of context compared to object categorization in our study (except Experiment 2), especially with increased vertical eccentricity, is in agreement with a coarse-to-fine model of visual processing^43^, where LSF, particularly important to recognize contexts, are processed earlier than HSF, which play a greater but not exclusive role in object recognition. However, both the finding that the objects were recognized in a considerable percentage of trials (approximately 30%-55%), at least when centered on the screen’s horizontal midline, and the influence of semantic consistency on object categorization outside the high-acuity central vision (whatever the specific image location) are in agreement with previous research showing object recognition without fixation^44–46^. Our study is, thus, also relevant for a crucial debate in the literature, where several investigations have argued, instead, that recognition of an object, to a point that activates knowledge about the probability of occurrence in a given scene, would happen only in central vision (upon fixation)^47,48^.

Finally, categorization of the objects and, to a lesser extent, the contexts varied considerably according to the image arrangement in the visual field, showing in particular a horizontal-vertical anisotropy^36,37^ with a huge performance decrease due to vertical eccentricity. This result highlights the need to carefully consider this dimension in any study of rapid visual categorization of objects and scene’s contexts.

## Conclusion

In five experiments, we showed that the facilitation of visual categorization of contexts and objects when they are semantically consistent compared to when they are inconsistent is a robust effect. Indeed, it emerges not only when the object is embedded in the context, belonging to the same image, but also when the object and the context are in separate images and in different locations in the visual field. Our results suggest that this effect is driven by percept-to-representation matching mechanisms, while it does not primarily act on early processing of visual features and extraction of the stimulus from its surroundings (contrary to what the perceptual schema model claims) and does not mainly depend on guessing (contrary to what the functional isolation model claims). Thus, our study supports the object selection model and, finding a mutual interplay between the object and the context, extends it to explain categorization of the scene’s context within the theoretical framework of an object-context reciprocal influence of matching processes (object-context selection account).

## General Method

### Participants

Eighty individuals, 16 different in each experiment (Experiments 1–4: 8 females each, Experiment 5: 14 females), volunteered for no remuneration. All reported normal or corrected-to-normal visual acuity, normal color vision and no history of neurological disorders. Participants were aged 18 to 41 years old, with mean age (*SD*): 27.5 (*6*.7), 26.6 (*5.1*), 31.1 (*5.6*), 27.4 (5.6), 24.1 (*4.4*), for Experiments 1–5, respectively. All were right handed, with mean% of laterality quotient (*SD*) at the Edinburgh Laterality Inventory^49^: 86 (7), 92 (*7*), 74 (31), 79 (24), 68 (31), for Experiments 1–5, respectively. This study was approved by the LAPCOS of the Université Côte d’Azur and carried out in accordance with the declaration of Helsinki. All participants gave informed written consent before participating.

### Stimulus material and design

Eighty full-color photographs of animated and unanimated objects, plus six as practice, and 80 full-color photographs of indoor and outdoor scene’s contexts, plus six as practice, were used. Objects were selected from Konkle *et al*.’s database^50^ or Google Images, contexts were selected from the SUN database^51^ or Google Images. “Object” images were defined by a unique object (e.g., cow, parasol). “Context” images were mainly defined by global, spatial information (e.g., mountain, beach), even though they also contained local information. Eight versions of each experimental scene used in Experiment 1 were created using Adobe Photoshop CS 9.0 (Adobe, San Jose, CA). First, a semantically consistent object was inserted within a context (e.g., the cow in the mountain, the parasol in the beach), starting between 2° and 3° horizontally from the context’s closest edge (the small variations of object placement were required to avoid spatial inconsistency within the context).

Second, to create an inconsistent version of each scene, two scene contexts were paired, and their consistent objects swapped (e.g., the cow was included in the beach and the parasol in the mountain; Fig. 1a). The pairs were formed by different combinations of animate or inanimate objects and outdoor or indoor contexts, in order to avoid that any results might be fundamentally driven by fast processing of specific visual features based on their distinctiveness. In other words, we aimed to avoid misinterpreting any effect due to strong visual differences between animate and inanimate objects^52^ or indoor and outdoor contexts^41^ as a semantic consistency effect. Therefore, semantically consistent object-context pairs were composed of 20 animate-outdoor, 12 animate-indoor, 20 inanimate-outdoor and 28 inanimate-indoor pairs; semantically inconsistent object-context pairs were composed of 13 animate-outdoor, 19 animate-indoor, 27 inanimate-outdoor and 21 inanimate-indoor pairs.

We repeated the first and second step inserting a mirror reversed object, to control for any effect of viewpoint typicality or eccentricity of its most diagnostic part (e.g., the handle in a cup). We then mirror reversed each scene to present it in each hemifield (left or right) with the object maintaining the same eccentricity from the central fixation point. For Experiments 2–5, the contexts and the objects were presented in separate images but paired and mirrored in the same way as in Experiment 1. Moreover, each object was pasted into a different colored 1/*f* noise background, created in Matlab R2015a (MathWorks, Inc., Natick, MA), which did not evoke any semantics. The objects had the same size as in Experiment 1. They were placed starting at 2° horizontally from the background’s closest edge and centered vertically in the background. We also created, in Matlab, a white noise mask. All experimental images and the mask were shown at 10° × 8° (315 × 252 pixels). Moreover, we created visual response cues. In Experiment 1, the cue was a small white rectangle (3.24° × 2.76°, 102 × 87 pixels) with a black outline, where a black rectangular region indicated the scene’s quadrant containing the object (this was done to prevent the participant to respond to another portion of the scene by mistake, thinking that it corresponded to the target object). In Experiments 2–5, the cue was a black arrow (2.5° × 1°, 79 × 32 pixels) pointing toward the screen’s region where the image to categorize first had been presented.

### Apparatus

The experiments were conducted on a HP Compaq dc7100 computer running OS Windows XP. The stimuli were presented on a DELL 7004 CRT screen (32.5 cm × 24.5 cm, 1024 × 768 pixels, refresh rate: 85 Hz). Stimulus presentation and response recording was controlled by Experiment Builder (SR Research, Canada). Eye movements were recorded using an EyeLink 1000 (SR Research, Canada) at a sampling rate of 1000 Hz. Viewing was binocular, but only the dominant eye was tracked. A chinrest stabilized the eyes approximately 57 cm away from the screen.

### Procedure

Participants were tested individually, seated in a quiet, dimly illuminated room, in front of the screen, and laying with their forehead and chin on the chinrest. Responses were given orally and transcribed by the experimenter. Prior to the experiment, participants underwent a randomized nine-point calibration procedure, which was validated to ensure that the average error was <0.5° and the maximum error in one of the calibration points <1°. Recalibrations were performed during the task if necessary. Before each trial sequence, a single-point calibration check was applied while the participant fixated a dot in the center of the screen. The screen background color was medium grey (RGB = 127,127,127). At the beginning of the experiment, participants performed six practice trials, followed by 80 experimental trials presented in random order. Each trial started with a 90-ms black central fixation cross (Times New Roman, bold, font size 40), followed by the experimental image(s) for 130 ms, an 80-ms mask and a 150-ms medium grey blank screen. After this, a central cue indicated which item (i.e., whether the context or the object) the participant had to name first and was displayed until response. Examples of the presentations of the experimental scenes, mask and response cue in each experiment are depicted in Fig. 2.

**Figure 4:**
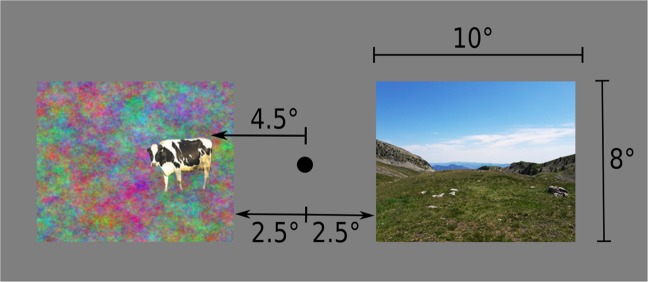
Image placement in Experiment 2. The images were placed at a horizontal eccentricity from the screen’s center of 2.5° considering the closest edge of the image. Consequently, the closest edge of the object was placed at 4.5° of horizontal eccentricity from the screen’s center. In Experiment 1 (object embedded in the context), only one image was presented in each trial, in the same location as one of the two images in Experiment 2. These images were not used as experimental materials and are presented here only for illustrative purposes. The image of mountain was adapted from photo taken by the first author. The image of the cow have been cropped from a larger picture which can be found at this link: https://search.creativecommons.org/photos/864c293f-7e2f-4ff8-b4be-9a76b261fc61.

In Experiment 1 (object embedded in the context) and Experiment 2 (object and context presented separately; Fig. 4) the images were centered on screen’s horizontal midline. In Experiment 3, the object image and the context image were presented in the same hemifield one above the other (one in the upper left quadrant and one in the lower left quadrant, or one in the upper right quadrant and one in the lower right quadrant). All images (closest edges) appeared at an eccentricity from the screen’s center of 2.5° horizontally and 2° vertically (therefore, the closest edge of each image was at 5.5° and the closest edge of the object to be categorized was at 7.5° overall eccentricity from the screen’s center). In Experiment 4, the images were presented each in a different hemifield, both above or below the screen’s horizontal midline (one in the left upper quadrant and one in the right upper quadrant, or one in the lower left quadrant and the other in the lower right quadrant), at the same eccentricity as in Experiment 3. In Experiment 5, the images were presented centered on the screen’s horizontal midline, each in a different hemifield as in Experiment 2, but at a greater eccentricity (the closest edge of each image was at 5.5° and the closest edge of the object to be categorized was at 7.5° from the screen’s center), to have the same overall distance from the screen’s center as in Experiments 3 and 4.

Each experimental image was presented only once during the experiment and all of the experimental factors were counterbalanced within- and between participants. Each experiment lasted approximately 40 minutes.

### Data analysis

All results were scored blind to experimental condition. Coding was cross validated by two independent coders considering correct any response that named the image at the entry level (e.g., dog) or the subordinate level (e.g., Bernese Bouvier). Synonyms at an equal level of descriptiveness (at the entry level; e.g., puppy) or labels indicating an incorrect subordinate level (e.g., Shetland Sheepdog) but belonging to the correct entry level were also scored as correct. Responses at a superordinate level (e.g., “animal”) were scored as incorrect (see^7,10^ for a similar scoring procedure). We removed from analysis all trials in which the error in the single-point calibration check was >1° or the distance of gaze position from the screen’s center was >1° when the experimental image(s) appeared, in order to ensure that participants maintained central fixation. Then intrusion responses (errors semantically consistent with the other, co-occurring image when this was correctly categorized, see^10^), were removed from the analyses (see Table 1 for a summary of data removals in each experiment).

All analyses (Generalize Linear Mixed Models, independent-samples *t* tests) were conducted on accuracy (binary response: correct or incorrect). GLMMs were run using the lmer() function of the lme4 package^53^ in the R programming environment (The R Foundation for Statistical Computing, Version 3.3.2); see Results for information about the models’ structure. For each model, we reported the predictors’ coefficients (*β*-values), *SE*-values, *z-*values, and associated *p*-values. Graphics were created using the ggplot2 package^54^. We used GLMMs because they have many advantages over traditional ANOVA models. They provide more statistical power considering all the trials without a priori grouping them into averages by subject and condition, and allow a simultaneous estimation of the variance due to subjects and items^55,56^. In addition, GLMMs are very robust to data unbalance between experimental conditions (e.g., because of removals), allowing analyses to be performed preserving statistical power^57^.

## Supplementary information

Supplementary Materials.

## Data Availability

The data from this study and the images used are available from the corresponding author on request.
