# Author Correction: Reciprocal semantic predictions drive categorization of scene contexts and objects even when they are separate

**DOI:** 10.1038/s41598-020-69046-3

**Published:** 2020-07-15

**Authors:** Anaïs Leroy, Sylvane Faure, Sara Spotorno

**Affiliations:** 1 Laboratoire d’Anthropologie et de Psychologie Cliniques, Cognitives et Sociales (LAPCOS), Université Cote d’Azur, Nice, France; 2 School of Psychology, University of Keele, Keele, United Kingdom

Correction to: * Scientific Reports* https://doi.org/10.1038/s41598-020-65158-y, published online 21 May 2020

In Figure 1b, the bottom right image shows a beach where it should show a mountain. The correct Figure 1 appears below.

**Figure 1:**
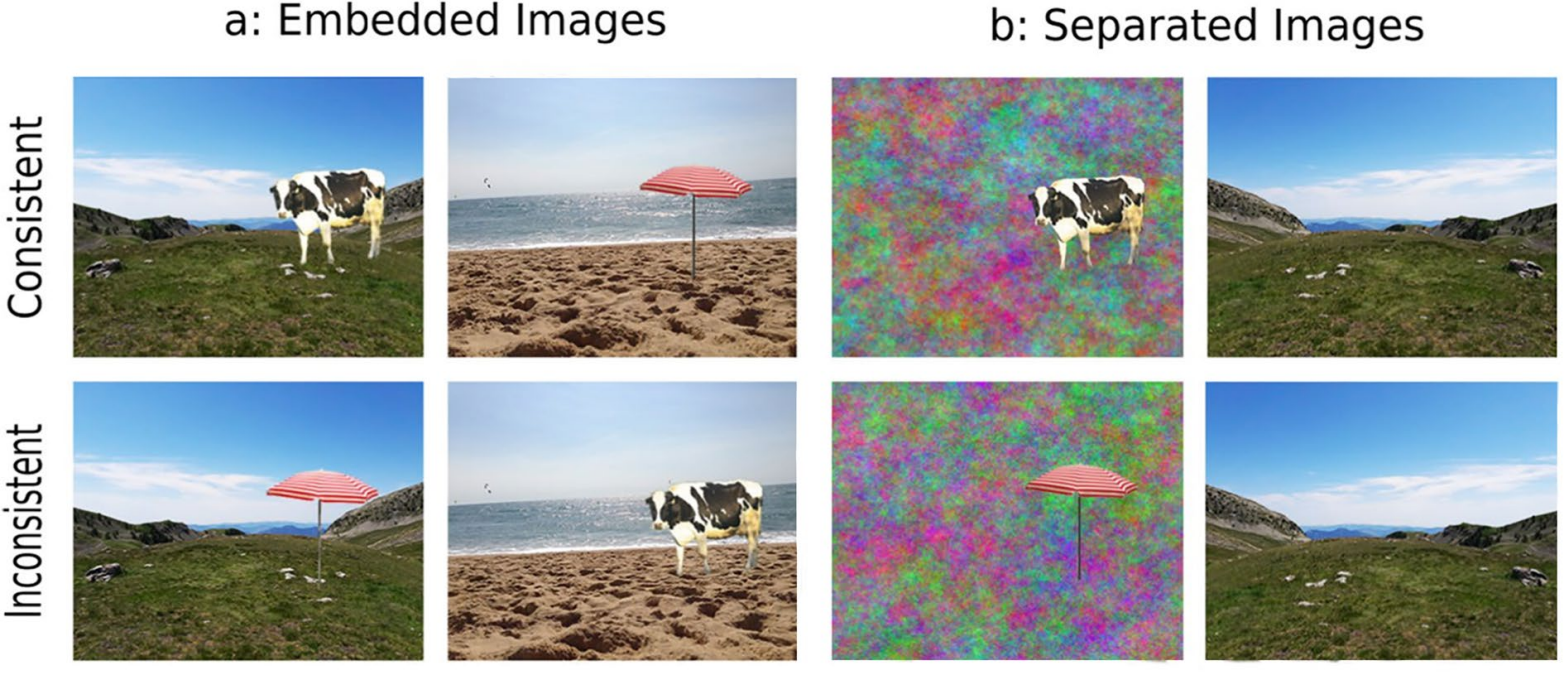
Examples of semantically consistent (top row) and inconsistent (bottom row) presentations, when either the object was included into the scene’s context (**a**: Experiment 1) or the object and the context were shown simultaneously but separately, with the object pasted onto a 1/*f* colored noise background (**b**: Experiments 2–5). The images of the mountain and of the parasol were adapted from photos taken by the first author, while the images of the beach and of the cow were adapted from photos shared in Creative Commons, under a CC BY-SA 2.0 license (https://creativecommons.org/licenses/by-sa/2.0/?ref=ccsearch&amp;atype=rich) for the beach and a CC BY 2.0 license (https://creativecommons.org/licenses/by/2.0/?ref=ccsearch&amp;atype=rich) for the cow. The image of the beach can be found at this link: https://search.creativecommons.org/photos/8668bad5-87ab-471b-a392-888595f28e96. The only change to this image was a mirror reversal to illustrate the visual presentation in each hemifield (see General Method for more details). The image of the cow have been cropped, and mirror reversed, from a larger picture which can be found at this link: https://search.creativecommons.org/photos/864c293f-7e2f-4ff8-b4be-9a76b261fc61. These images were not used as experimental materials and are presented here only for illustrative purposes.

In addition, a typesetting error led to the following sentence being incorrectly displayed as a sub-heading in the Introduction. It should be read as plain text.

“Different outcomes would support different theories”

