# Supplementary material for: Reciprocal semantic predictions drive categorization of scene contexts and objects even when they are separate
Source: Sci Rep. 2020 May 21;10:8447. doi: 10.1038/s41598-020-65158-y (PMC7242336; doi:10.1038/s41598-020-65158-y)
Supplement: Supplementary file 1 — Supplementary Materials. [file 41598_2020_65158_MOESM1_ESM.pdf]

Supplementary Materials

Reciprocal semantic predictions drive categorization of scene contexts and objects even when they are separate

Anaïs Leroy, Sylvane Faure, Sara Spotorno

Supplementary Table S1

Results of the GLMMs on accuracy (binary response: correct or incorrect), carried out for each experiment including trials with intrusion responses. Each model included Semantic Association (Consistent vs. Inconsistent), Image Type (Context vs. Object) and their interaction as predictors, and participants and images as random factors. All models had full random structure. For each model, we report the predictors' coefficients (*β*-values), the *SE*-values, the *z*-values, and the associated *p*-values.

**Results with intrusion response trials**				
**Experiment 1**				
	β	*SE*	*z*	*p*
Semantic Association	-1.373	0.223	-6.151	<0.001
Image Type	-0.854	0.284	-3.004	0.003
Interaction	-0.247	0.389	-0.635	0.525
**Experiment 2**				
	β	*SE*	*z*	*p*
Semantic Association	-0.954	0.222	-4.308	<0.001
Image Type	-0.364	0.297	-1.224	0.221
Interaction	-0.219	0.472	-0.464	0.643
**Experiment 3**				
	β	*SE*	*z*	*p*
Semantic Association	-0.368	0.178	-2.073	0.038
Image Type	-2.62	0.356	-7.369	<0.001
Interaction	-0.509	0.358	-1.423	0.155
**Experiment 4**				
	β	*SE*	*z*	*p*
Semantic Association	-0.719	0.160	-4.484	<0.001
Image Type	-2.227	0.310	-7.176	<0.001
Interaction	-0.227	0.341	-0.667	0.505
**Experiment 5**				
	β	*SE*	*z*	*p*
Semantic Association	-0.978	0.179	-5.475	<0.001
Image Type	-1.089	0.268	-4.062	<0.001
Interaction	-0.489	0.344	-1.424	0.154

Supplementary Table S2

Results of the GLMMs for the corrected accuracy (e.g., hit rate minus intrusion), carried out for each experiment. Each model included Semantic Association (Consistent vs. Inconsistent), Image Type (Context vs. Object) and their interaction as predictors, and participant and image as random factors. For each model, we report the predictors' coefficients (β-values), the *SE*-values, the *z*-values, and the associated *p*-values. Models for experiments 1, 2 and 5 had full random structure. Models for Experiment 3 and 4 are described below:

Model for Experiment 3:

Corrected Accuracy ~ Image Type * Semantic Association + (1+Image Type || Scenes) + (1+Image Type || Subject)

Model for Experiment 4:

Corrected Accuracy ~ Image Type*Semantic Association + (1+Image Type:Semantic Association || Scene) + (1+Image Type:Semantic Association || Subject)

**Corrected accuracy (hit rate minus intrusion rate)**				
**Experiment 1**				
	β	*SE*	*z*	*p*
Semantic Association	-0.932	0.121	-7.692	<0.001
Image Type	-0.601	0.166	-3.626	<0.001
Interaction	-0.285	0.262	-1.089	0.276
**Experiment 2**				
	β	*SE*	*z*	*p*
Semantic Association	-0.632	0.130	-4.872	<0.001
Image Type	-0.228	0.172	-1.322	0.186
Interaction	-0.115	0.262	-0.439	0.661
**Experiment 3**				
	β	*SE*	*z*	*p*
Semantic Association	-0.271	0.114	-2.376	0.018
Image Type	-1.947	0.186	-10.451	<0.001
Interaction	-0.427	0.229	-1.865	0.062
**Experiment 4**				
	β	*SE*	*z*	*p*
Semantic Association	-0.541	0.109	-4.983	<0.001
Image Type	-1.577	0.109	-14.507	<0.001
Interaction	-0.361	0.270	-1.338	0.181
**Experiment 5**				
	β	*SE*	*z*	*p*
Semantic Association	-0.691	0.112	-6.185	<0.001
Image Type	-0.765	0.143	-5.351	<0.001
Interaction	-0.420	0.211	-1.991	0.047
